# A Modified Switching Procedure from Temporary to Tunneled Central Venous Dialysis Catheters

**DOI:** 10.3390/jcm13123367

**Published:** 2024-06-07

**Authors:** Johannes Eberhard, Constantin Bedau, Andrew Genius Chapple, Julia Klein, Christoph Reissfelder, Anna-Isabelle Kaelsch, Andreas Lutz Heinrich Gerken, Sebastian Zach, Kay Schwenke

**Affiliations:** 1 Department of Surgery, University Medical Center Mannheim, University of Heidelberg, 69117 Mannheim, Germany; 2 Biostatistics Core, Department of Interdisciplinary Oncology, School of Medicine, LSU Health Sciences Center, New Orleans, LA 70112-7021, USA; 3 Vth Department of Medicine (Nephrology/Endocrinology/Rheumatology), University Medical Center Mannheim, University of Heidelberg, 69117 Mannheim, Germany

**Keywords:** hemodialysis, catheter, tunneled catheter, temporary catheter, guidewire exchange, infection

## Abstract

**Background:** Tunneled central venous catheters are commonly used for dialysis in patients without a functional permanent vascular access. In an emergent setting, a non-tunneled, temporary central venous catheter is often placed for immediate dialysis. The most critical step in the catheter insertion is venipuncture, which is often a major cause for longer intervention times and procedure-related adverse events. To avoid this critical step when placing a more permanent tunneled catheter, an exchange over a previously placed temporary one can be considered. In this paper, we present a modified switching approach with a separate access site. **Methods**: In this retrospective analysis of a prospective database, we examined whether this modified technique is non-inferior to a de novo application. Therefore, we included all 396 patients who received their first tunneled dialysis catheter at our site from March 2018 to March 2023. Out of these, 143 patients received the modified approach and 253 the standard de novo ultrasound-guided puncture and insertion. Then, the outcomes of the two groups, including adverse events and infections, were compared by nonparametric tests and multivariable logistic regression. **Results:** In both groups, the implantations were 100% successful. Catheter explantation due to infection according to CDC criteria was necessary in 18 cases, with no difference between the groups (5.0% vs. 4.4% *p* = 0.80). The infection rate per 100 days was 0.113 vs. 0.106 in the control group, with a comparable spectrum of bacteria. A total of 12 catheters (3 vs. 9) had to be removed due to a periinterventional complication. An early-onset infection was the reason in two cases (1.3%) in the study group and five in the control group (1.9%). A total misplacement of the catheter occurred in two cases only in the control group. After adjustment for potential confounders via multivariable logistic regression there was not a significant difference in the complication rate (adjusted odds ratio, aOR = 0.53, 95% CI = 0.14–2.03, *p* = 0.351) but an estimated decreased risk overall based on the average treatment effect of −1.7% in favor of the study group. **Conclusions:** The present study shows that a catheter exchange leads to no more infections than a de novo placement; hence, it is a feasible method. Moreover, misplacements and control chest X-rays to exclude pneumothorax after venipuncture were completely avoided by exchanging. This approach yields a much lower infection rate than previous reports: 1.3% compared to 2.7% in all existing aggregated studies. The presented approach seems to be superior to existing switching methods. Overall, an exchange can also help to preserve veins for future access, since the same jugular vein is used.

## 1. Introduction

Tunneled central venous catheters (TCVCs) are commonly used for hemodialysis in patients with end-stage renal disease (ESRD) without a working arteriovenous fistula/graft or dysfunction of an already existing vascular access. In acute kidney failure, a temporary (i.e., non-tunneled) dialysis catheter (CVC) is often placed when the patient needs urgent dialysis. If the patient still requires hemodialysis for more than 2 weeks, a TCVC should be placed according to the guidelines [1]. This happens in about 24% of cases [2].

TCVCs-related complications can be divided into periinterventional and delayed complications [3]. The most critical step in the insertion procedure is the correct puncture of the vein. A difficult puncture of the vein is often a major cause for a longer intervention time and procedure-related adverse events [4]. A faulty puncture of the artery can lead to hematoma, arteriovenous fistula, bleeding from the orifice, an air embolism or a pneumothorax in up to 2.1% of all implantations [4,5,6]. Other periinterventional complications include mechanical complications such as kinking or cardiac complications like arrhythmias, valvular damage or pericardial effusion [7]. To avoid this critical step, several authors have reported an exchange over an existing catheter to be equal to a de novo placement [2,8,9,10].

After successful placement, 30–56% of catheters develop at least one complication during the catheter’s retention time. These often require a replacement and therefore contribute significantly to morbidity [11,12]. Possible complications include catheter-related bloodstream infections, (central) venous stenosis [13] and clotting. Catheter thrombosis can lead to malfunction and pulmonary embolism. Further, a broad range of mechanical complications can occur, such as catheter displacement or rupture, with consecutive bleeding or air embolism [7]. Numerous studies investigated the factors influencing TCVC complications. They showed that complication risk is related to the time span between placement and removal and patient history of TCVCs [4]. The prevalence of TCVC infections is correlated with the number of comorbidities [11,14,15]. Similarly, it was shown that higher mortality rates in CVC patients could be attributed to patient-related factors and comorbidities [15,16]. Diverging results were found between patient characteristics and central vein stenosis [13]. All previous authors of an exchange used the same venotomy site for both catheters and some reported higher short-term infection rates for the study group (i.e., exchange group); however, these were not considered significant [8].

With the intention of reducing the risk of infection, we present a modified approach with a separate venotomy site. A similar technique was used in a study by LeClaire et al. for an exchange of a TCVC with promising results [17].

The goal of the present study was to determine whether this modified technique is equivalent to a new application with a venous puncture (non-inferiority) in both periinterventional and long-term complications, especially with regard to catheter-associated infections.

## 2. Materials and Methods

In this retrospective analysis of our prospective database, we identified all patients over the age of 18 receiving their first TCVC between March 2018 and March 2023 (*n* = 396) at a German university tertiary medical center.

The study was approved by the local ethics committee (MA 2023-879). All TCVCs were implanted in jugular veins, as this is the preferred site and is generally recommended [3].

Baseline patient characteristics such as age, gender and underlying renal disease were recorded. Comorbidities such as hypertension, diabetes, peripheral artery disease, congestive heart failure, arterial fibrillation, coronary heart disease, active cancer and history of stroke, as well as specific risk factors like additional artificial material at the timepoint of TCVC implantation and implantation side, were recorded.

Complications were defined as those requiring surgical explantation of the CVC and determined to be related to the CVC. These were subdivided into dislocation, dysfunction and early- (<30 days) or late-onset (>30 days) infection. An infection was defined according to the CDC as clinical manifestations and at least 1 positive blood culture from a peripheral vein and no other apparent source, with either a positive semiquantitative (>15 Colony-forming units (CFU)/catheter segment) or quantitative (>102 CFU/catheter segment) culture, whereby the same organism (species and antibiogram) is isolated from the catheter segment and a peripheral blood sample, or simultaneous quantitative cultures of blood samples with a ratio of ≥3:1 (catheter vs. peripheral) or a differential period of catheter culture versus peripheral blood culture positivity of 2 h [1,18].

### 2.1. Procedure

All insertions were conducted in an operational theater by or under the direct supervision of a board-certified vascular surgeon under sterile conditions and local anesthesia. All patients received prophylactic antibiotics. In the control group, implantation was performed according to the usual standards. Venipuncture of the jugular vein was performed under ultrasound guidance.

**Figure 1:**
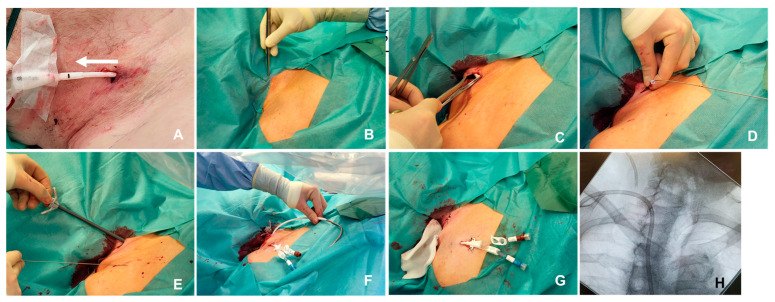
Step-by-step pictures of the modified procedure (**A**): The temporary CVC is pulled back slightly and fixated. (**B**): The old CVC is completely clear of the sterile field; the insertion is denoted by forceps. (**C**): Through a separate incision, the old catheter is dissected. (**D**): A guidewire is inserted and the old catheter is removed. (**E**): Insertion of a peel-away sheath. (**F**): Tunneling of the TCVC. (**G**) Completed procedure. (**H**): X-ray control with both the old and new catheters.

The experimental group procedure is explained step by step:

Step 1: The existing temporary CVC is released from its fixation and pulled back approximately 3 cm to ensure that the potentially unsterile part of the exposed catheter is retracted from the exit site (cf. Figure 1A).

Step 2: The surgical site is disinfected and sterile drapes are applied according to general standards. The existing catheter exit site is hereby covered by drapes to ensure sterile conditions for the placement of the new TCVC (cf. Figure 1B).

Step 3: The new ca. 1 cm incision is made 2–3 cm proximal to the CVC exit site, where the catheter can still be palpated under the skin within the sternocleidomastoid muscle (cf. Figure 1C). Step 4: The catheter is then exposed at the incision site, secured by a clamp and cut 2 cm above the new skin exit-point. A guidewire is inserted into the cut CVC under fluoroscopy and the old catheter is removed (cf. Figure 1D).

Step 5: The incision site is gradually dilated, a sheath is inserted and the new catheter (Medtronic, Palindrome) is implanted under the guidance of fluoroscopy.

The subsequent procedure was similar in both groups. A subcutaneous tunnel was prepared, the catheter was pulled through and its function was checked by injection and aspiration. This was followed by fluoroscopy for positioning and the incision was then closed using the Donati technique. If there was no resistance to the injection and aspiration, and the tip of the TCVC was correctly placed in the atrium, the operation was deemed successful.

An X-ray examination was performed within 24 h to rule out a pneumothorax after venipuncture in the control group.

### 2.2. Statistics

R statistical software version 4.3.0 was used for all analyses. Categorical variables were summarized by reporting counts and percentage, while continuous variables were summarized by reporting the mean and standard deviation. Categorical variables were compared across groups using Fisher’s exact test, while continuous variables were compared using the Wilcoxon rank sum test. Multivariable logistic regression was used to adjust for potential confounding variables (age, gender, number of comorbidities and implantation side) and determine the effect of the experimental procedure on complication risk. Estimated average treatment effects (ATE) were computed by averaging the predicted probabilities of complications with and without each factor and calculating their difference. The ATE for the study group can be interpreted as the estimated adjusted change in complication rate for patients if they were in the study group instead of the control group. A *p*-value < 0.05 was considered statistically significant.

## 3. Results

The study included 396 patients, with the experimental group consisting of 96 (67.1%) men and 47 (32.9%) women and the control group consisting of 165 (65.2%) men and 88 (34.8%) women. Table 1 displays the descriptive characteristics of the population separated by treatment group. There was no significant difference between the groups with regard to demographic data, the side of the body of implantation and the proportion of individuals with foreign bodies in central venous vessels.

Across all kidney conditions, there was not a significant difference across the study groups (*p* = 0.7). However, there was a significantly higher amount of hepatorenal syndrome in the study group (9.78% vs. 2.4%, *p* = 0.003). All other kidney conditions occurred in similar frequencies between the two groups. Patients in both groups had comparable comorbidities (2.64 average vs. 2.80, *p* = 0.449) and there was no difference in specific complication rates (all *p* > 0.13). The proportion of people with pre-existing conditions was also comparable in both groups, with the greatest difference between patients with peripheral arterial occlusive disease (Table 1). In both groups, the implantations were 100% successful.

A total of 70 patients experienced an adverse event which required surgical revision. Out of these, 12 events were considered to be a periinterventional complication. Table 2 displays the adverse events by study group.

In 35 cases, the catheter was explanted due to a suspected clinical infection; in 18 of these cases, the infection was confirmed according to the CDC definition. In the study group, there was a total of seven infections (5%). The mean time between implantation and explantation was 148 days. In the control group, 11 patients had a confirmed catheter-associated infection; the time between implantation and explantation was 152 days. There was no statistically significant difference between the groups in terms of these values (cf. Table 2). The infection rate per 100 days was 0.113 in the study group and 0.106 in the control group. The microbiologic spectrum of the infections was similar.

Early-onset infection rates were comparable (1.3% vs. 1.9%), while the mean time to infection was lower in the control group without reaching significance (22 days vs. 13 days *p* = 0.07). A mechanical dysfunction, such as kinking, was the reason in a comparable number of cases (one vs. two, *p* = 1.00). A total misplacement of the catheter occurred in two cases in the control group but did not happen in the study group. Differences in the periinterventional complications were not significantly different across the groups.

The occurrence of long-term adverse events, such as thrombosis and dislocation requiring a new catheter, also lacked a significant difference.

If patients are grouped by the occurrence of complications including infections (complication: 31 patients and no complication: 365 patients) independent of the experimental and control group, there is no significant difference in the distribution according to gender, mean age, implantation side, leading kidney diseases and previous illnesses.

**Figure 2:**
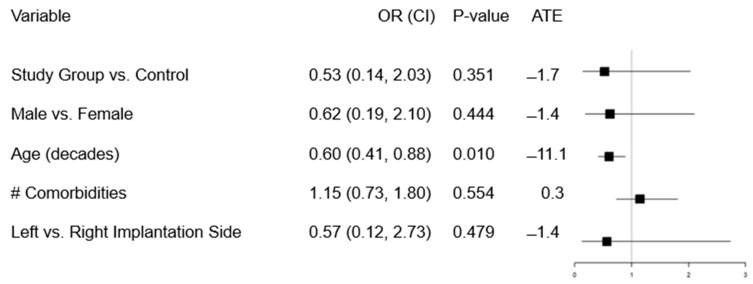
Multivariable logistic regression results predicting complications. Odds ratios (OR) and *p*-values are reported, in addition to average treatment effects (i.e., absolute adjusted predicted increase in risk of infection/complication) due to each factor.

Figure 2 displays the results of the multivariable logistic regression, predicting any complications. After adjusting for gender, age, implantation side and number of comorbidities, there was no significant difference in the complication rate for the study vs. the control group (adjusted odds ratio, aOR = 0.53, 95% CI = 0.14–2.03, *p* = 0.351). There was, however, an estimated decreased risk overall based on the average treatment effect of −1.7%. Increased age was associated with a decreased complication risk (*p* = 0.01, aOR = 0.6, 95% CI = 0.41–0.88) but no other factors were significant.

## 4. Discussion

Providing working vascular access without complications is crucial for patients with ESRD.

In this study, we showed that in concordance with previous studies, an exchange is not inferior to a de novo placement [2,8,9,10]. Misplacements, which are a rare but possibly deadly complication, were completely avoided by exchanging an existing catheter. In our analysis, misplacements only occurred in the control group. The exchange can also help to preserve veins for future access, which is a crucial point for dialysis patients.

The clear limitation of our analysis is its retrospective and non-randomized design. But bias was limited by exclusively using the new method when a CVC was present and documenting almost 400 consecutive cases.

In this study, complications were only recorded if they required a later explantation of the catheter. Complications arising only due to a puncture, and hence only occurring in the control group, such as a puncture of the artery, hematoma or pneumothorax, were not recorded and would also possibly favor the switching method [19]. Post-operative X-ray controls were only needed in the control group due to the puncture. This radiation could be completely avoided by our proposed method. Besides being as least as safe as a de novo placement, switching over an existing catheter is by far more feasible and less time-consuming, as the time-consuming step of venipuncture is skipped. This is becoming more important, as time in the operating theater is very costly and health care providers are increasingly forced to work as cost-effectively as possible.

Although there is no evidence of this in clinical practice, it is often perceived that a catheter exchange is associated with more infections as the old catheter could be considered contaminated. The modification we present in this manuscript aims to leave the part of the catheter that has been outside of the patient outside of the sterile field. Hence, the possible advantage of the presented approach could be lower infection rates compared to traditional switches over the existing skin exit-site. Overall, we perceive that the closer infection occurs to the implantation, the more likely it is to be related to the insertion. After a period of longer than 30 days, if the infection has not become clinically overt it is more likely that the infection is due to other causes such as improper handling or insufficient hygiene during dialysis. On the other hand, limiting periprocedural infections to 24 h, as some authors suggest, is too short a time period for a minor contamination to become clinically overt.

Compared to studies that report short-term infections, our approach yields a much lower infection rate of 1.3% compared to the 9.4% of Falk et al. and the bigger cohort of Bajaj et al. reporting 4.3% [8,10]. However, our overall short-term infection rate is also lower than in these studies. Van Ha et al. reported 30-day infections in a 97-patient cohort without a control group [9]. Our long-term infection rate of 0.11 per 100 catheter days is comparable to Van Ha and lower than the other publications [2,8,9,10], and can be judged as very good using the methodology of Beathard et al. [20]. When aggregated, all the studies yield a 2.7% 30-day infection rate, which is twice as high as our method. Hence, we believe that our approach reduces the risk of infection compared to classical switching methods. Our germ spectrum shows a microbiology that is consistent with previous reports [20]. Since this analysis is limited due to differences in the methodology of the studies, further studies should be carried out to elucidate this.

There have been conflicting reports about whether a short-term infection is significantly influenced by the duration of use of the CVCs [2,10]. Future studies should investigate this further.

Interestingly, infection was confirmed in only 51% of cases where a suspected clinical infection led to the explantation of the catheter. Taking into account that a patient loses his dialysis access and that a new application is fraught with complications, a more detailed diagnosis should always be carried out.

In conclusion, whenever possible, we prefer the hereby described exchange method because it is as safe as a de novo placement and significantly more feasible.

## 5. Conclusions

According to our data, the implantation of a TCVC via an existing CVC is a safe method. In comparison, we did not observe any increased infection or complication rates. This alternative procedure avoids additional venipuncture with its risks and protects the venous vessels. We also offer comprehensive data on both the exchange and de novo placement of TCVCs.

The practical advice and key step we want to emphasize is securing the utmost sterile conditions for catheter placement to prevent catheter infection. This is achieved by creating a small new incision and covering the whole existing CVC, including its exit site, with drapes.

Further studies using a prospective and randomized design to compare time savings, avoidance of post-operative X-ray and financial outlay, as well as complications during implantation, should be considered.

## Figures and Tables

**Table 1:** Patient demographics.

Variable	All (*n* = 396, %)	Study Group (*n* = 143, %)	Control (*n* = 253, %)	*p*-Value
**Sex**				
Male	261 (65.9)	96 (67.1)	165 (65.2)	0.741
Female	135 (34.1)	47 (32.9)	88 (34.8)	
**Age**				
Mean (SD)	68.08 (15.36)	66.81 (15.53)	68.79 (15.25)	0.216
**Implantation Side**				
Left	84 (21.2)	26 (18.2)	58 (22.9)	0.307
Right	312 (78.8)	117 (81.8)	195 (77.1)	
**Foreign Material**				
No	315 (79.5)	114 (79.7)	201 (79.4)	1
Yes	81 (20.5)	29 (20.3)	52 (20.6)	
**Underlying Renal Disease**				
Cardiorenal syndrome	40 (10.1)	13 (9.1)	27 (10.7)	0.729
Diabetic nephropathy	44 (11.1)	15 (10.5)	29 (11.5)	0.868
Hepatorenal syndrome	20 (5.1)	14 (9.8)	6 (2.4)	0.003
Hypertensive nephrosclerosis	44 (11.1)	17 (11.9)	27 (10.7)	0.741
Inflammatory	59 (14.9)	23 (16.1)	36 (14.2)	0.660
Malignant	23 (5.8)	10 (7)	13 (5.1)	0.504
Multifactorial	52 (13.1)	18 (12.6)	34 (13.4)	0.878
Others	43 (10.9)	16 (11.2)	27 (10.7)	0.868
Polycystic kidney disease	14 (3.5)	2 (1.4)	12 (4.7)	0.096
Toxic	10 (2.5)	4 (2.8)	6 (2.4)	0.752
Unknown	47 (11.9)	11 (7.7)	36 (14.2)	0.074
Comorbidities *				
Mean count (SD)	2.74 (1.55)	2.64 (1.54)	2.8 (1.56)	0.449
Hypertension	332 (83.8)	117 (81.8)	215 (85)	0.477
AFIB	117 (29.6)	39 (27.3)	78 (31)	0.492
CHD	149 (37.7)	51 (35.7)	98 (38.9)	0.589
Active Tumor	56 (14.2)	23 (16.1)	33 (13.1)	0.454
PAD	71 (18)	20 (14)	51 (20.2)	0.135
Stroke	39 (9.9)	15 (10.5)	24 (9.5)	0.861
Diabetes	183 (46.3)	66 (46.2)	117 (46.4)	1
HF	137 (34.7)	46 (32.2)	91 (36.1)	0.444

* One patient in the control group had missing comorbidity information. SD—standard deviation, AFIB—atrial fibrillation, CHD—coronary heart disease, PAD—peripheral artery disease, HF—heart failure.

**Table 2:** Adverse events by group.

	Study Group (*n* = 143, %)	Control (*n* = 253, %)	*p*-Value
**Infections**			
Overall infections	7 (5.0)	11 (4.4)	0.805
Mean time to explantation (d)	148.6	152.7	0.285
Infections with Staphylococci	5 (3.5)	9 (3.6)	1.00
Infections with Enterococci	1 (0.7)	0 (0)	0.361
Infections with Enterobacteriaceae	2 (1.4)	3 (1.2)	1.00
Infections with Candida	0 (0)	1 (0.4)	1.00
**Periinterventional complications**			
Overall	3 (2.1)	9 (3.6)	0.549
Infection within 30 days	2 (1.3)	5 (1.9)	0.659
- Mean time until infection (d) (SD)	21.5 (2.12)	13 (3.81)	0.079
Mechanical dysfunction	1 (0.7)	2 (0.8)	1.00
Misplacement	0 (0%)	2 (0.8)	0.537
**Long-term complications**			
Thrombosis	4 (2.8)	5 (2)	0.728
Accidental dislocation	9 (6.3)	12 (4.7)	0.495

## Data Availability

Dataset available on request from the authors.
